# Necrotizing Enterocolitis in an Infant With a History of Twin-Twin Transfusion Syndrome: A Case Report

**DOI:** 10.7759/cureus.56720

**Published:** 2024-03-22

**Authors:** Nga N Tran, Sydney Hutto, James Liu, Tyler Bullock, Richard Virgilio, David L Flowers

**Affiliations:** 1 Medical School, Edward Via College of Osteopathic Medicine, Auburn, USA; 2 Medical School, Kirksville College of Osteopathic Medicine, Kirksville, USA; 3 Nursing, Columbus State University, Columbus, USA; 4 Clinical Affairs, Edward Via College of Osteopathic Medicine, Auburn, USA; 5 Pediatric Medicine, Piedmont Columbus Regional Hospital Midtown Campus, Georgia, USA

**Keywords:** bloody diarrhea, premature infants, im-peds, twin twin transfusion, necrotizing enterocolitis (nec)

## Abstract

This case report describes necrotizing enterocolitis (NEC) in an infant with a history of twin-twin transfusion syndrome (TTTS). TTTS is a volume imbalance where the anastomosis at the vascular equator between the two placentae shifts from the donor to the recipient twin. This causes a higher risk for NEC, a marked inflammation caused by bacterial infection into the intestinal wall, from prematurity and intestinal hypoperfusion. Complications include sepsis, bowel necrosis, perforation, peritonitis, and death. NEC is a leading cause of morbidity in preterm infants.

A 3-month-old female with a history of TTTS and prematurity presented with her mother to the pediatric emergency department (ED) for bloody diarrhea, emesis, lack of appetite, and lethargy for 4 days. The pediatrician changed the formula due to a possible milk allergy, however, she continued to have bloody diarrhea. Over the 2 days, the patient had nonbilious and non-bloody emesis and couldn’t tolerate oral intake.

In the ED, labs showed neutropenia and sepsis. She had a positive fecal occult blood test (FOBT) and an abdominal x-ray that revealed dilated loops of bowel and pneumatosis intestinalis. She was started on intravenous (IV) fluids for maintenance of hydration. She was started on broad-spectrum antibiotics including intravenous (IV) vancomycin and meropenem, and had her feedings temporarily stopped. The patient was transferred to the pediatric intensive care unit (PICU) at a tertiary care/children's hospital that evening where she had a laparotomy performed to resect the diseased intestine. She was discharged 10 days after the surgery for home recovery with clinical follow-up.

## Introduction

Necrotizing enterocolitis (NEC) is marked inflammation and bacterial infection of the intestines, ranging from epithelial injury to transmural injury and perforation [1]. NEC is caused by bacterial invasion into the intestine wall in which an endotoxin binds to Toll-like receptor 4 (TLR4) triggering pathogen-associated molecular pattern (PAMP) receptors that break the intestinal barrier allowing the bacteria to translocate [1]. NEC is a leading cause of morbidity in preterm infants [1]. Bell’s classification rates the severity of NEC based on clinical and radiographic factors in the early progression of the disease [2]. Risk factors for NEC include formula feeding, intestinal dysbiosis, low birth weight, and prematurity [3]. The symptoms of NEC are non-specific and can include fatigue, decreased activity, decreased appetite, vomiting, diarrhea, increased abdominal girth, and blood in the stool [2]. The diagnostic test for NEC is an abdominal plain radiograph series anterior-posterior (AP) and left lateral decubitus plain views showing loops of bowel and pneumatosis intestinalis [2]. NEC is treated with intravenous (IV) broad-spectrum antibiotics, a nasogastric tube for decompression of dilated bowel, and a nothing-by-mouth (NPO) diet [2]. Laparotomy is indicated for severe symptoms, such as bowel perforation, with worsening symptoms, or in patients who do not respond to medical therapy [2].

Twin-twin transfusion syndrome (TTTS) is a volume imbalance across vascular anastomoses between monochorionic twins [4]. TTTS is the predominant cause of previable pregnancy loss in monochorionic twins; therefore these pregnancies should be monitored with ultrasounds every two weeks after 16 weeks gestation until birth [4]. The anastomosis at the vascular equator between the two placentae shifts from the donor to the recipient twin [5]. Complications include growth restriction in the donor twin, cardiomyopathies in recipients, and neurodevelopmental morbidities in the survivor [6]. Quintero staging is based on the appearance of the donor fetus’ bladder, abnormal umbilical blood flow, the presence of hydrops, or the death of one twin, and ranges from stage I to V [7]. There are two treatment options for TTTS: amnioreduction (AR) or fetoscopic laser surgery (FLS) [8,9]. FLS is the first-line treatment, as it reduces both mortality and morbidity [9].

Studies show that twins with TTTS are more susceptible to developing NEC and isolated small bowel perforation [10]. This increased risk is attributed to prematurity and reduced blood flow to the intestines [11]. The compromised blood flow leads to hypoxic damage, necrosis, and perforation of the gastrointestinal wall, which causes complications like septicemia and death [12]. Therefore, it is crucial to closely monitor and promptly treat twins with TTTS to mitigate the risk of developing NEC.

## Case presentation

A 3-month-old female with a history of twin-twin transfusion syndrome (TTTS) and prematurity presented with her mother to the pediatric emergency department (ED) for evaluation and treatment of bloody diarrhea, emesis, lack of appetite, and lethargy for 4 days. The mother stated that she noticed her daughter had specks of bloody diarrhea, so she took the patient to the pediatrician who changed the patient’s formula due to possible milk allergy. However, the patient continued to have multiple episodes of bloody diarrhea despite changing the formula. Over the 2 days prior to her presentation to the ED, she had non-bilious and non-bloody emesis and couldn’t tolerate oral intake. Her mother stated that she had episodes of choking and increased work of breathing after eating.

**Figure 1:**
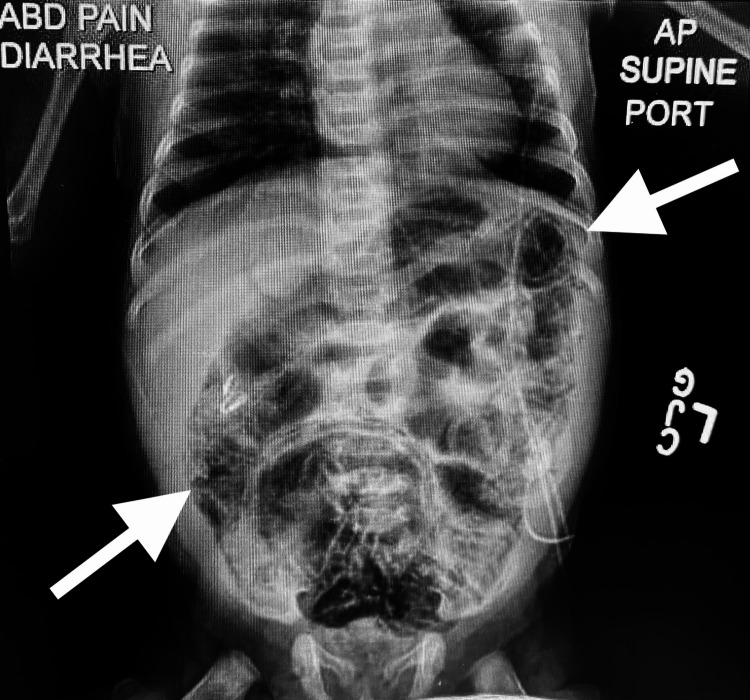
Abdominal x-ray demonstrates dilated loops of bowel and pneumatosis

Upon arrival to the ED, the patient’s vital signs were as follows: blood pressure (BP) 86/64 mmHg, pulse 192 beats per minute, temperature 99.3°F, respiratory rate 50 breaths per minute, length 17.72 inches, weight 3.36 kg, body mass index (BMI) 16.59 kg/m2, and SpO2 100%. Her physical exam showed pale skin, tachycardia, abdominal distension with guarding and hypoactive bowel sounds, a diaper rash, and bloody diarrhea mixed with foul-smelling stool. The labs were drawn in the ED with results of thrombocytosis due to inflammation, high hemoglobin and hematocrit due to hemoconcentration, elevated procalcitonin due to sepsis, low white blood cell count (WBC) and neutropenia due to sepsis which is summarized in Table 1. A fecal occult blood test (FOBT) was positive while the blood and stool cultures were negative. An x-ray of the abdomen demonstrated dilated loops of the bowel and pneumatosis intestinalis (Figure 1).

**Table 1:** Laboratory results from the Pediatric Emergency Department (ED)

Test Name	Lab Value	Normal Reference
White Blood Cell Count	4.80	6.0-17.5 x 10^3^/µL
Red Blood Cell Count	4.81	2.7-4.5 x 10^6^/µL
Hemoglobin	14.5	9.5-13.5 g/dL
Hematocrit	41.7	29-41%
Platelets	566	150,000-450,000/µL
Procalcitonin	0.77	<0.05 ng/mL
Lactic Acid	2.24	1.0-3.3 mmol/L
C-Reactive Protein	1.02	<10 mg/L

In the ED, she was started on intravenous (IV) fluids for maintenance of hydration. She was started on broad-spectrum antibiotics including IV vancomycin and meropenem, and had her feedings temporarily stopped. Due to neutropenia and sepsis, the patient was placed on hemodynamic monitoring, which included blood pressure, temperature, respiratory status, and heart rate every 15 minutes. The patient was transferred to the pediatric intensive care unit (PICU) at a tertiary care/children's hospital that evening where she had a laparotomy to resect the grossly diseased intestine. She was discharged 10 days after the surgery for home recovery with clinical follow-up. 

## Discussion

NEC typically manifests within the first two weeks of life with symptoms of bloody stool and abdominal distention [13]. NEC is caused by inflammation and damage to the intestinal tissue, affecting the intestinal lining [1]. The condition arises in premature infants due to their underdeveloped digestive system. Prematurity is a major risk factor, and in the case of our patient, a history of TTTS also increases the risk of developing NEC. TTTS is a condition that specifically affects identical twins who share a placenta [4]. The shared placenta acts as the lifeline for both twins, supplying them with essential nutrients and oxygen through a network of blood vessels. An imbalanced distribution of blood flow results when the blood vessels within the placenta become connected in an abnormal manner. Consequently, the recipient twin receives a surplus of blood, while the donor twin experiences a reduced blood supply. Although our patient presented with “classic” signs and symptoms, it is uncommon for an infant to develop these complications outside of the first month of life [3].

Our patient received the standard treatment for NEC including lab tests, IV fluids, and antibiotics. Surgery for bowel resection can be a difficult recovery for infants with NEC [13]; with our patient being 3 months old, it’s possible that gastrointestinal development could have allowed her to recover from the surgery better than a neonate. The need for surgery can be a large financial cost and the access to a tertiary facility where the surgery can be performed are barriers to care that may present challenges to parents [13].

Early assessment and diagnosis are critical to getting a patient with NEC the surgical intervention as fast as possible, so they will have the best chance for a successful outcome and recovery [13]. The recommendation for referral for our patient to seek emergency care and evaluation could have possibly been given sooner, as opposed to the two weeks of formula changes. Feeding intolerance in an infant is common, but the length of time in combination with bloody stools should signal prompt medical evaluation. This illustrates the need for more watchful assessments for conditions such as NEC so that treatment can be delivered promptly.

Preventive measures play a crucial role in minimizing the risk and impact of the two significant health conditions affecting newborns, NEC and TTTS. For NEC, promoting breastfeeding and avoiding the early introduction of formula feeding can be beneficial. Implementing strategies such as probiotic supplementation and strict infection control measures in neonatal intensive care units (NICUs) can also help prevent NEC [12]. Early identification through regular prenatal ultrasound examinations is essential. Timely intervention, such as laser ablation or amnioreduction, can be employed to address the unequal blood flow between twins in the placenta, reducing the risk of adverse outcomes. These preventive measures hold great promise in safeguarding the health and well-being of newborns affected by NEC and TTTS.

## Conclusions

This case report documented an occurrence of NEC in an infant with a history of TTTS. The case highlighted the increased risk of NEC in infants with TTTS, emphasizing the need for increased awareness and early intervention. The association between TTTS and NEC is believed to be due to prematurity, intestinal hypoperfusion, and bacterial infection, which can lead to inflammation and complications such as sepsis, bowel necrosis, perforation, peritonitis, and death. The findings illustrate the need for early recognition, aggressive management, and potential surgical intervention in severe cases of NEC in infants with TTTS. Further research is needed to enhance our understanding of the mechanisms involved, and to delineate additional preventative and treatment strategies.

## References

[1] Ginglen JG, Butki N (2024 Jan-). Necrotizing enterocolitis. StatPearls [Internet].

[2] Alganabi M, Lee C, Bindi E, Li B, Pierro A (2019). Recent advances in understanding necrotizing enterocolitis. F1000Res.

[3] Thänert R, Keen EC, Dantas G, Warner BB, Tarr PI (2021). Necrotizing enterocolitis and the microbiome: current status and future directions. J Infect Dis.

[4] Miller JL (2021). Twin to twin transfusion syndrome. Transl Pediatr.

[5] Twin-to-Twin transfusion Syndrome (TTTS). (2021 (2021). Twin-to-Twin Transfusion Syndrome (TTTS) | Johns Hopkins Medicine. https://www.hopkinsmedicine.org/health/conditions-and-diseases/twintotwin-transfusion-syndrome-ttts.

[6] Wagner S, Repke JT, Ural SH (2013). Overview and long-term outcomes of patients born with twin-to-twin transfusion syndrome. Rev Obstet Gynecol.

[7] National Guideline Alliance (UK) (2019 Sep). Evidence Review for Ultrasound Screening for Feto-Fetal Transfusion Syndrome: Twin and Triplet Pregnancy: Evidence Review A.. London: NICE.

[8] Ovesen P, Rasmussen S, Kesmodel U (2011). Effect of prepregnancy maternal overweight and obesity on pregnancy outcome. Obstet Gynecol.

[9] Schifsky K, Deavenport-Saman A, Mamey MR (2021). Risk factors for parenting stress in parents of children treated with laser surgery for twin-twin transfusion syndrome 2 years postpartum. Am J Perinatol.

[10] Detlefsen B, Boemers TM, Schimke C (2008). Necrotizing enterocolitis in premature twins with twin-to-twin transfusion syndrome. Eur J Pediatr Surg.

[11] Chiang MC, Lien R, Chao AS, Chou YH, En Chen YJ (2003). Clinical consequences of twin-to-twin transfusion. Eur J Pediatr.

[12] Philip I, Ford A, Haslam R (2002). Congenital bowel perforation in twin-to-twin transfusion syndrome. Pediatr Surg Int.

[13] Neu J, Walker WA (2011). Necrotizing enterocolitis. N Engl J Med.

